# Ascertaining an Appropriate Diagnostic Algorithm Using EGFR Mutation-Specific Antibodies to Detect EGFR Status in Non-Small-Cell Lung Cancer

**DOI:** 10.1371/journal.pone.0059183

**Published:** 2013-03-11

**Authors:** Guiyang Jiang, Chuifeng Fan, Xiupeng Zhang, Qianze Dong, Liang Wang, Yang Liu, Shundong Dai, Lianhe Yang, Yong Zhang, Juanhan Yu, Enhua Wang

**Affiliations:** Department of Pathology, First Affiliated Hospital and College of Basic Medical Sciences, China Medical University, Shenyang, China; Sapporo Medical University, Japan

## Abstract

**Background:**

Epidermal growth factor receptor (EGFR) mutation status is the most valuable indicator in the screening of non-small-cell lung cancer (NSCLC) patients for tyrosine kinase inhibitor (TKI) therapy. Accurate, rapid and economical methods of detecting EGFR mutations have become important. The use of two mutation-specific antibodies targeting the delE746-A750 mutation in exon 19 and L858R mutation in exon 21 makes this task possible, but the lack of consensually acceptable criteria for positive results limits the application of this antibody based mutation detection.

**Methods:**

We collected 399 specimens from NSCLC patients (145 resection specimens, 220 biopsy specimens, and 34 cytology specimens) whose EGFR mutation status had been detected by TaqMan PCR assay. Immunohistochemical (IHC) analyses using EGFR mutation-specific antibodies were employed for all samples. After staining and scoring, the sensitivity, specificity, positive predictive value (PPV) and negative predictive value (NPV) were calculated in accordance with different levels of positive grades in comparison with the results of PCR-based assay.

**Results:**

In IHC-based analyses, 144 cases were scored 0, 104 cases were scored 1+, 103 cases were scored 2+, and 48 cases were scored 3+. With the molecular-based results were set as the “gold standard”, the prevalence of mutation was 6.94% (10/144), 23.08% (24/104), 67.96% (70/103) and 100% (48/48), respectively, for samples with scores 0, 1+, 2+ and 3+. When score 3+ was considered positive, the specificity and PPV were 100%; if only score 0 was considered negative, 93.06% NPV was obtained.

**Conclusion:**

Patients with score 3+ have a perfect PPV (100%), and may accept TKI treatment directly without any molecular-based assays. Patients with score 0 had high NPV (93.06%), which could reach 97.22% when the detection of total EGFR was applied. However, samples with score 1+ or 2+ are unreliable and need further verification of EGFR mutation status by molecular-based assays.

## Introduction

Since the beginning of use of the epidermal growth factor receptor tyrosine kinase inhibitors (EGFR-TKIs) gefitinib and erlotinib for treatment of advanced non-small-cell lung cancer (NSCLC) [1], studies have shown that NSCLC patients with EGFR-activating mutations can benefit from TKI treatment [2], [3]. The status of EGFR mutations has become the best predictor of the response to TKIs [4]–[9]. Direct sequencing is the “gold standard” method for the detection of EGFR mutations. However, its sensitivity is relatively low; if the percentage of tumor cells is <25%, the probability of a false-negative result is greatly increased. Due to lack of a sufficient number of tumor cells available for extraction of high-quality DNA, the probability of obtaining a false-negative is increased while testing a small biopsy or cytology sample. However, about 70% of lung cancers are diagnosed at advanced stages whereby small biopsies and cytological specimens are the only source of material for the diagnosis and mutation testing. Recent advances in molecular methods have enabled a development of more sensitive methods for detecting mutations. Such methods include real-time quantitative polymerase chain reaction (qRT-PCR) using specific probes (TaqMan PCR assay), amplified refractory mutation system (ARMS) and high-resolution melting analysis (HRMA) [10]–[14]. However, they are expensive and invariably require good experimental conditions and sophisticated instruments. Hence, they are rarely applied in non-teaching hospitals.

Immunohistochemical (IHC) analyses can also be used to screen for EGFR mutations. Yu et al. developed EGFR mutation-specific rabbit monoclonal antibodies against EGFR with the E746–A750 deletion in exon 19 or the L858R point mutation which showed good consistency compared with molecular-based assays [15]–[26]. However, the practical application of this method was severely limited because of the appreciable difference in the criteria used for positive results among the different research teams. Some researchers scored data according to staining intensity, and divided samples into four grades: 0, 1+, 2+ and 3+. However, some authors advocated a score above 1+ to be considered positive,and others even argued that a score above 2+ should be scored positive. The specificities of these two approaches were relatively close (96–100%), but their sensitivities varied widely (47–92%) [15]–[19]. Some researchers also obtained a score for expression by multiplying the staining intensity by the percentage of staining area (0–300 or 400), and respectively advocated a score of >10 or >20 to be categorized as positive, but an appreciable difference in sensitivities (42.2–100%) and specificities (77–100%) was observed [20]–[22]. Recently, Kawahara et al. proposed that immunostaining should be classified as positive (score of 2+), negative (score of 0) or equivocal (score of 1+), indicating questionable, negative or weak expression, respectively, which can obtain a sensitivity of 81.4%, specificity of 97.5%, positive predictive value (PPV) of 94.6%, and negative predictive value (NPV) of 90.6% [23]. Consensus of a universally accepted criterion for “positive” is lacking, which severely hinders the clinical use of EGFR mutation-specific antibody to detect EGFR mutations.

In the present study, we analyzed the IHC results of 399 samples of NSCLC patients which were scored by a four-grading method taking into account the intensity and area of staining. We then compared the results with a molecular-based assay to explore the possibility of screening for EGFR mutations in NSCLC samples by IHC analyses.

## Materials and Methods

### Ethics Statement

All human tissues and cells were obtained in accordance with Human Subject Research Protocols approved by the China Medical University Review Board. Tumor tissues and cells were obtained with written informed consent from adult patients with NSCLC.

### Specimen selection and the molecular-based assay

We collected 399 specimens (145 resection specimens, 220 biopsy specimens, and 34 cytology specimens) from NSCLC patients who had applied for the molecular-based assay of EGFR mutations in the Department of Pathology of China Medical University (Shenyang, China) from August 2008 to August 2012. The age range of the lung cancer patients was 26–89 years (median, 62 years); there were 214 men and 185 women. The histological types were 341 adenocarcinomas, 47 squamous cell carcinomas, and 11 other types (Table 1). The EGFR mutation status of each specimen was detected by the TaqMan PCR assay. Resection and biopsy tissues were fixed in 10% formalin and embedded in paraffin. The supernatants of pleural-effusion specimens were removed by centrifugation, and the remaining cellular components were then fixed in 10% formalin and embedded in paraffin. The paraffin blocks were cut at a thickness of 8 µm for investigation of PCR-based EGFR gene mutations. Mutations of the EGFR gene were examined in exons 19 and 21 using the TaqMan PCR assay. Genomic DNA from paraffin-embedded tissue or cytology samples was extracted and purified using a QIAamp DNA Micro kit (Qiagen, Valencia, CA, USA). The primers for mutation detection and TaqMan probes targeting E746–A750 and L747–P753insS deletion mutations in exon 19,and L858R and L861Q point mutations in exon 21 were purchased from GP Medical Technologies (Beijing, China). The TaqMan PCR assay was carried out using a 7900HT Real-time PCR system (Applied Biosystems, Foster, CA, USA).

**Table 1:** Clinicopathological characteristics in subjects with lung cancer.

	Total (n = 399)	Resection (n = 145)	Biopsy (n = 220)	Cytology (n = 34)
**Age (average)**	61.7	60.2	62.7	61.7
**Sex**				
Male	214	70	126	18
Female	185	75	94	16
**Histology**				
Squamous cell carcinoma	47	15	32	0
Adenocarcinoma	341	127	188	26
Other types	11	3	0	8

### IHC analyses

Paraffin blocks were cut to a thickness of 4 µm for immunostaining. Conventional de-waxing and hydration treatment were undertaken using xylene and a graded series of ethanol, respectively. Specimens were boiled in a water-bath at 100°C for 20 min in 1 mmol/L ethylenediamine tetra-acetic acid (EDTA) at pH 9.0 and target retrieval solution (Dako, Glostrup, Denmark) to recover antigens. Intrinsic peroxidase activity was blocked by treatment with peroxidase blocking reagent (Dako) for 15 min at room temperature. The non-specific binding points were blocked by incubating with normal nonimmune goat serum for 15 min at room temperature. After washing in Tris-buffered saline (TBS; Dako) for 15 min, three primary antibodies (i.e., EGFR monoclonal antibody (D38B1), delE746–A750 mutation-specific monoclonal antibody (6B6) and L858R mutation-specific monoclonal antibody (43B2); Cell Signaling Technology, Danvers, MA, USA) were diluted separately at 1∶400 and added to the specimens. Specimens were incubated at 4°C overnight, washed in TBS for 15 min, and incubated with labeled polymer-horseradish peroxidase secondary antibody (ChemMate Envision kit; DAKO) for 30 min at room temperature. After washing in TBS for 15 min, the slides were visualized using 3,3′-diaminobenzidine.

### IHC scoring

The IHC staining score was based on the staining intensity and percentage of staining area in the membrane and/or cytoplasm of tumor cell. Four grades were employed: 0, 1+, 2+, 3+. Zero denoted no staining; 1+ denoted light yellow staining with no obvious particulates or yellow staining with obvious particulates in <10% of tumor cells; 2+ denoted yellow staining with obvious particulates in >10% tumor cells or brown staining with obvious particulates in <10% of tumor cells; and 3+ denoted brown staining with obvious particulates in >10% tumor cells. Staining assessment was carried out only if >5 tumor cells were present in a needle biopsy or cytology specimen. All immunohistochemical analyses were evaluated by three experienced investigators (YL, JHY and YZ) who were unaware of patients' clinical conditions or pathologic diagnosis.

### Statistical analyses

The sensitivity, specificity, PPV and NPV of the IHC-based assay were calculated using the molecular-based assay as a reference. The agreement between the 2 techniques was calculated using Cohen κ. All data were analyzed using SPSS 13.0 for Windows.

## Results

### Molecular-based EGFR mutational status of 399 NSCLC samples

A total of 399 NSCLC specimens were detected using the TaqMan PCR assay. An EGFR mutation was detected in 162 cases (40.6%, 162/399). This included an E19 (E746–A750) deletion mutation in 86 cases (21.55%, 86/399), E21 (L858R) point mutations in 70 cases (17.54%, 70/399), both E746–A750 and L858R mutations in 4 cases, E19 (L747–P753insS) deletion mutations in 4 cases (1.03%, 4/399),E21 (L861Q) point mutations in 6 cases (1.5%, 6/399), and no mutation in 237 cases (59.4%, 237/399). The rate of mutations in resection specimens was 40.69% (59/145), in biopsy specimens was 36.82% (81/220), and in cytology specimens was 35.29% (12/34).

### Comparative analyses of IHC-based and molecular-based EGFR mutational status

**Figure 1:**
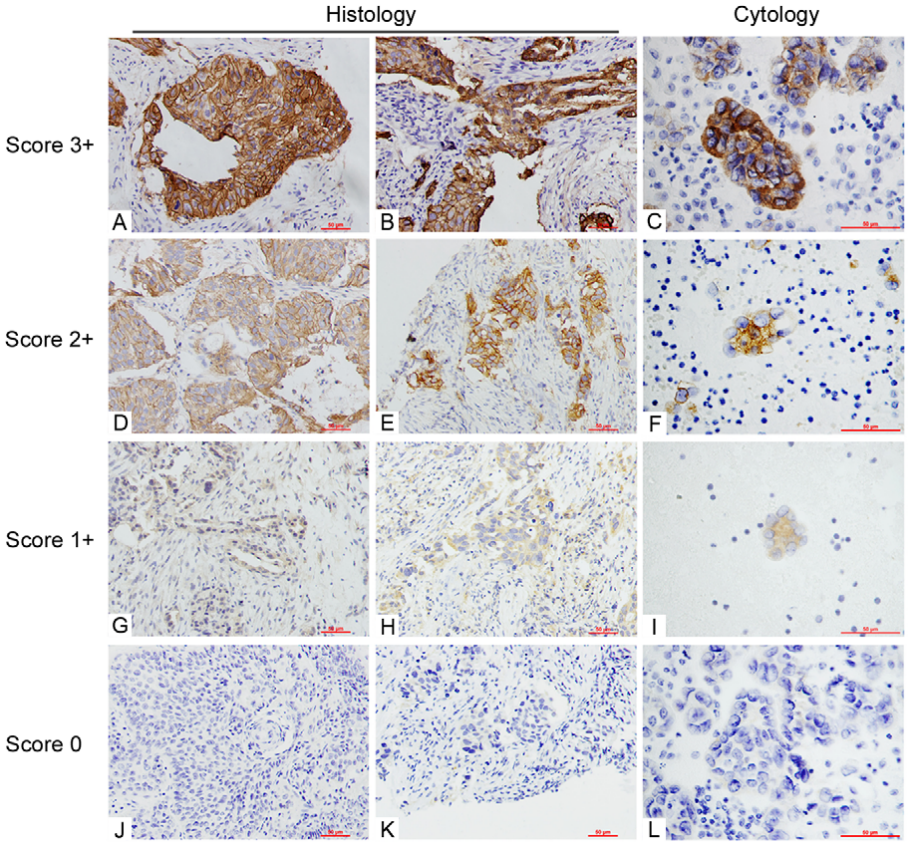
Staining of EGFR mutation-specific antibodies. Representative immunostaining of both histology and cytology samples in NSCLC patients (**A**, **D**, **G** and **J**, resection specimens, 200×; **B**, **E**, **H** and **K**, biopsy specimens, 200×; **C**, **F**, **I** and **L**, cytology specimens, 400×). Four grades were employed: 0, 1+, 2+ and 3+. 3+ denoted brown staining with obvious particulates in >10% tumor cells (**A**, **B** and **C**); 2+ denoted yellow staining with obvious particulates in >10% tumor cells or brown staining with obvious particulates in <10% of tumor cells (**D**, **E** and **F**); 1+ denoted light yellow staining with no obvious particulates or yellow staining with obvious particulates in <10% of tumor cells (**G**, **H** and **I**); Zero denoted no staining (**J**, **K** and **L**).

The staining patterns of EGFR mutation-specific antibody are shown in Figure 1. Out of 399 NSCLC specimens, 144 cases were scored 0 (in which 10 cases harbored EGFR mutations and the rate of mutations in this category was 6.94%, 10/144); 104 cases were scored 1+ (there were 24 cases of EGFR mutations and the rate of mutation was 23.08%, 24/104); 103 cases were scored 2+ (EGFR mutations were present in 70 cases and the rate of mutations was 67.96%, 70/103); 48 cases were scored 3+ (48 cases harbored EGFR mutations, and the rate of mutation was 100%, 48/48). Detailed results are shown in Table 2. The samples scored 3+ have a perfect PPV (100%), and those scored 0 have a good NPV (93.06%).

**Table 2:** Comparative analyses of IHC-based and molecular-based EGFR mutational status.

IHC-based assay		Molecular-based assay (TaqMan PCR assay)			
		Mutation+			Mutations-
Score	Numbers	delE746–A750	L858R	Mutation	Or
				prevalence	other mutations
0	144	3	7	6.94%(10/144)	134(93.06%)
Resection	52	1	1	3.85%(2/52)	50(96.15%)
Biopsy	77	1	5	7.79%(6/77)	71(92.21%)
Cytology	15	1	1	13.33%(2/15)	13(86.67%)
1+	104	14	10	23.08%(24/104)	80(76.92%)
Resection	40	8	2	25%(10/40)	30(75%)
Biopsy	55	5	5	18.18%(10/55)	45(81.82%)
Cytology	9	1	3	44.44%(4/9)	5(55.56%)
2+	103	40	31	67.96%(70*/103)	33(32.04%)
Resection	35	14	16	82.56%(29*/35)	6(17.14%)
Biopsy	60	24	13	61.67%(37/60)	23(38.33%)
Cytology	8	2	2	50%(4/8)	4(50%)
3+	48	29	22	100%(48*/48)	0(0)
Resection	18	10	8	100%(18/18)	0(0)
Biopsy	28	18	13	100%(28*/28)	0(0)
Cytology	2	1	1	100%(2/2)	0(0)
Total	399	86	70	38.1% (152*/399)	247(61.9%)

* :The cases with both E19 (E746–A750) and E21 (L858R) mutations were not counted twice.

Other mutations:L747–P753insS deletion mutation in exon 19 and L861Q point mutation in exon 21.

The agreement between the IHC-based and molecular-based assays in accordance with different levels of positive grades was calculated using Cohen κ. When the groups with score of 0 or 1+ were considered as negative, and those with score of 2+ or 3+ were considered as positive, the agreement between the 2 different detection methods was highest (κ = 0.644). However, there would be 24 false-negative cases (23.08%, 24/104) in the group of score 1+ and 33 false-positive cases (32.04%, 33/103) in that of score 2+, resulting in a sensitivity of 77.63%, specificity of 86.64%, PPV of 78.14%, and NPV of 86.4% for the detection by immunohistochemistry. None of these values was ideal (shown in Table 3).

**Table 3:** Comparative analyses of the IHC-based results on resection, biopsy and cytology specimens.

Judgment regarding positive result	Type	Sensitivity (%)	Specificity (%)	PPV (%)	NPV (%)	κ
	Total	93.42	54.25	55.69	93.06	0.422
= 0 as negative	Resection	96.61	58.14	61.29	96.15	0502
≥1 as positive	Biopsy	92.59	51.08	52.45	92.21	0.335
	Cytology	83.33	59.09	52.63	86.67	0.181
	Total	77.63	86.64	78.14	86.4	0.644
≤1 as negative	Resection	79.66	93.02	88.68	86.96	0.739
≥2 as positive	Biopsy	80.25	83.45	73.86	87.89	0.626
	Cytology	50	81.82	60	75	0.331
	Total	31.58	100	100	70.37	0.364
≤2 as negative	Resection	30.51	100	100	67.72	0.342
= 3 as positive	Biopsy	34.57	100	100	72.4	0.4
	Cytology	16.67	100	100	68.75	0.206

With molecular testing as a standard, the delE746–A750 mutation-specific antibody (6B6) could detect 69 of the 86 cases with an E746–A750 deletion mutation (40 with score 2+ and 29 with score 3+) whereas it was negative in the remaining 17 cases (4 with score 0 and 14 with score 1+) (κ = 0.584, sensitivity: 80.23%, specificity: 85.3%, PPV: 60%, NPV: 94.01%); the L858R mutation-specific antibody (43B2) could identify 53 of 70 cases with a L858R point mutation (31 with score 2+ and 22 with 3+), whereas it was negative in the remaining 17 cases (7 with score 0 and 10 with score 1+) (κ = 0.639, sensitivity: 75.71%, specificity: 91.79%, PPV: 66.25%, NPV: 94.67%).

Additionally, we also detected total EGFR in all 399 cases using monoclonal antibody against EGFR. The results showed 27 cases were scored 0, 38 cases were scored 1+, 193 cases were scored 2+, and 141 cases were scored 3+. EGFR monoclonal antibody (D38B1) is different from the two mutation-specific antibodies. DelE746–A750 mutation-specific antibody (6B6) can specifically recognize the EGFR proteins with an E746–A750 deletion mutation in exon 19, and L858R mutation-specific antibody (43B2) is able to specifically identify the EGFR protein with a L858R point mutation in exon 21; in contrast, EGFR monoclonal antibody (D38B1), which is not a mutation-specific antibody, identifies the total EGFR protein regardless of the mutation status. Although total EGFR was highly expressed in most cases, there were still 38 cases with score 1+ and 27 cases with score 0, in which 12 cases were tested positive for EGFR gene mutations by molecular method. This may be explained by the fact that the levels of total EGFR in tumor cells are so low, that the staining of the two mutation-specific antibodies may be negative in some cases, even though they have EGFR gene mutations detected (as shown in Figure S1). Therefore, detecting the level of total EGFR using the EGFR monoclonal antibody (D38B1) could prevent the emergence of similar false-negative results above, while the sensitivities of delE746–A750 and L858R mutation-specific antibodies could be increased to 82.56% and 90% respectively.

### Comparative analysis of the IHC-based results of resection, biopsy and cytology specimens

**Figure 2:**
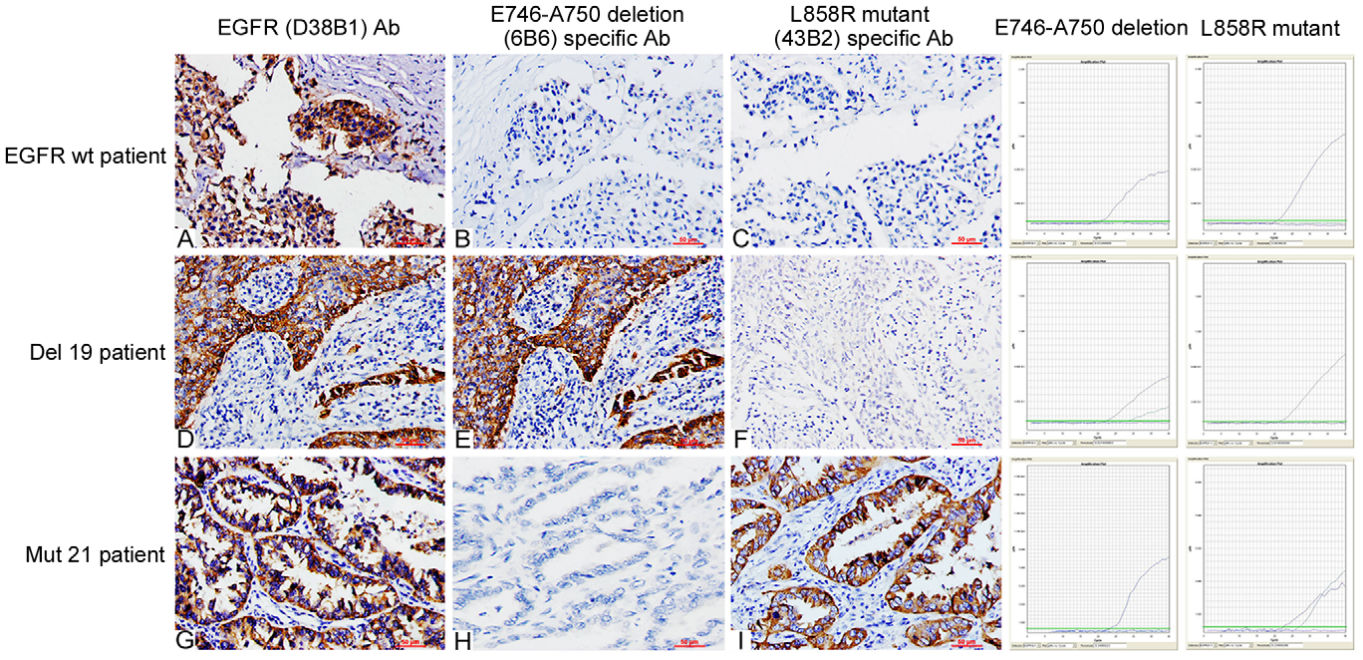
Staining of resection specimens using EGFR mutation-specific antibodies. The total EGFR protein in resection samples could be stained with EGFR (D38B1) antibody (**A**, **D** and **G**, 200×). Samples without EGFR mutations were not stained with two mutation-specific antibodies (**B** and **C**, 200×). Samples with E746–A750 deletion mutation were stained with E746–A750 deletion (6B6) specific antibody (**E**, 200×) and samples with L858R mutation were stained with L858R mutant (43B2) specific antibody (**I**, 200×).

The staining patterns of resection specimens using EGFR mutation-specific antibodies were shown in Figure 2. According to our scoring criteria, of the 145 resection specimens, 52 were scored 0, in which 2 cases harbored EGFR mutations and the mutation rate was only 3.85% (2/52); 40 cases were scored 1+, in which there were 10 cases of EGFR mutations and the mutation rate was 25% (10/40); 35 cases were scored 2+, in which EGFR mutations were present in 29 cases, and the mutation rate was 82.56% (29/35); 18 cases were scored 3+, in which 18 cases harbored EGFR mutations, and the mutation rate was 100% (18/18). The detailed results were shown in Table 2.

**Figure 3:**
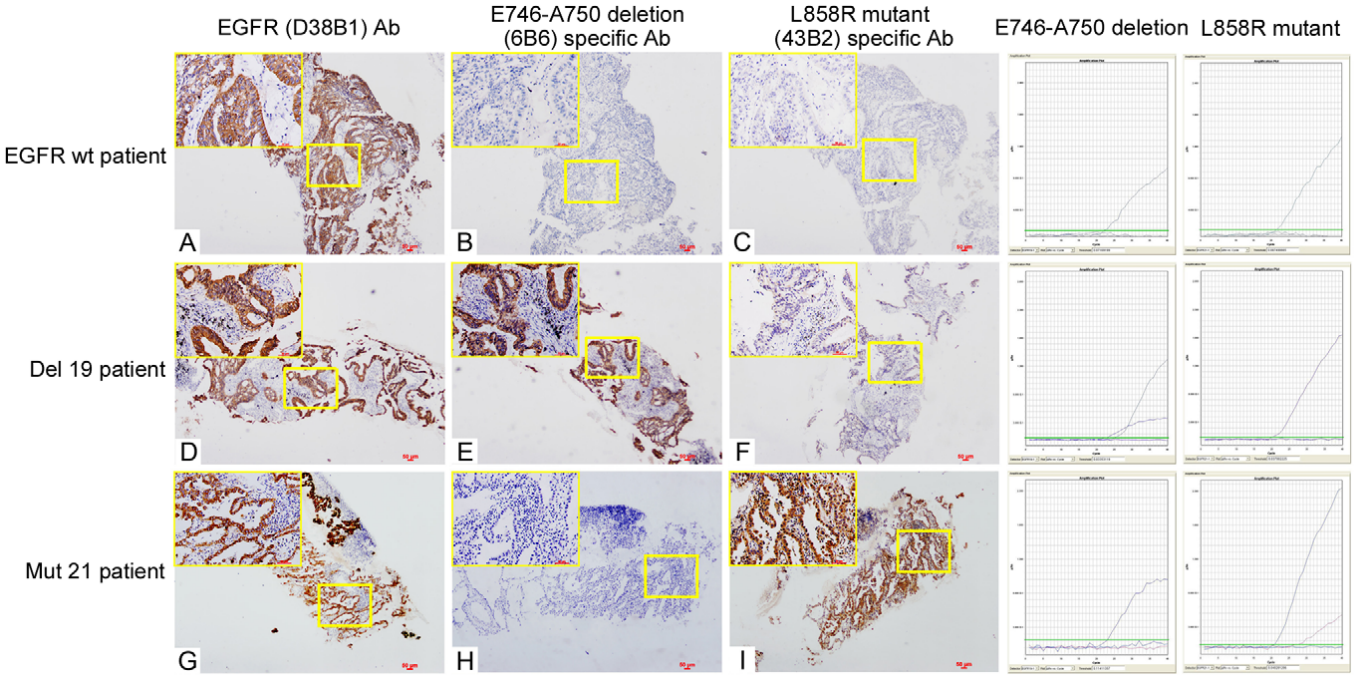
Staining of biopsy specimens using EGFR mutation-specific antibodies. The total EGFR protein in biopsy samples could be stained with EGFR (D38B1) antibody (**A**, **D** and **G**, 40× and the upper left corner 200×). Samples without EGFR mutations were not stained with two mutation-specific antibodies (**B** and **C**, 40× and the upper left corner 200×). Samples with E746–A750 deletion mutation were stained with E746–A750 deletion (6B6) specific antibody (**E**, 40× and the upper left corner 200×) and samples with L858R mutation were stained with L858R mutant (43B2) specific antibody (**I**, 40× and the upper left corner 200×).

The staining features of biopsy specimens using EGFR mutation-specific antibodies were shown in Figure 3. According to our scoring criteria, of the 220 resection specimens, 77 cases were scored 0, in which 6 harbored EGFR mutations and the mutation rate was 7.79% (6/77); 55 cases were scored 1+, in which 10 cases harbored EGFR mutations and the mutation rate was 18.18% (10/55); 60 cases were scored 2+, in which 37 harbored EGFR mutations, and the mutation rate was 61.67% (37/60); 28 cases were scored 3+, in which 28 cases harbored EGFR mutations, and the mutation rate was 100% (28/28). The detailed results were shown in Table 2.

**Figure 4:**
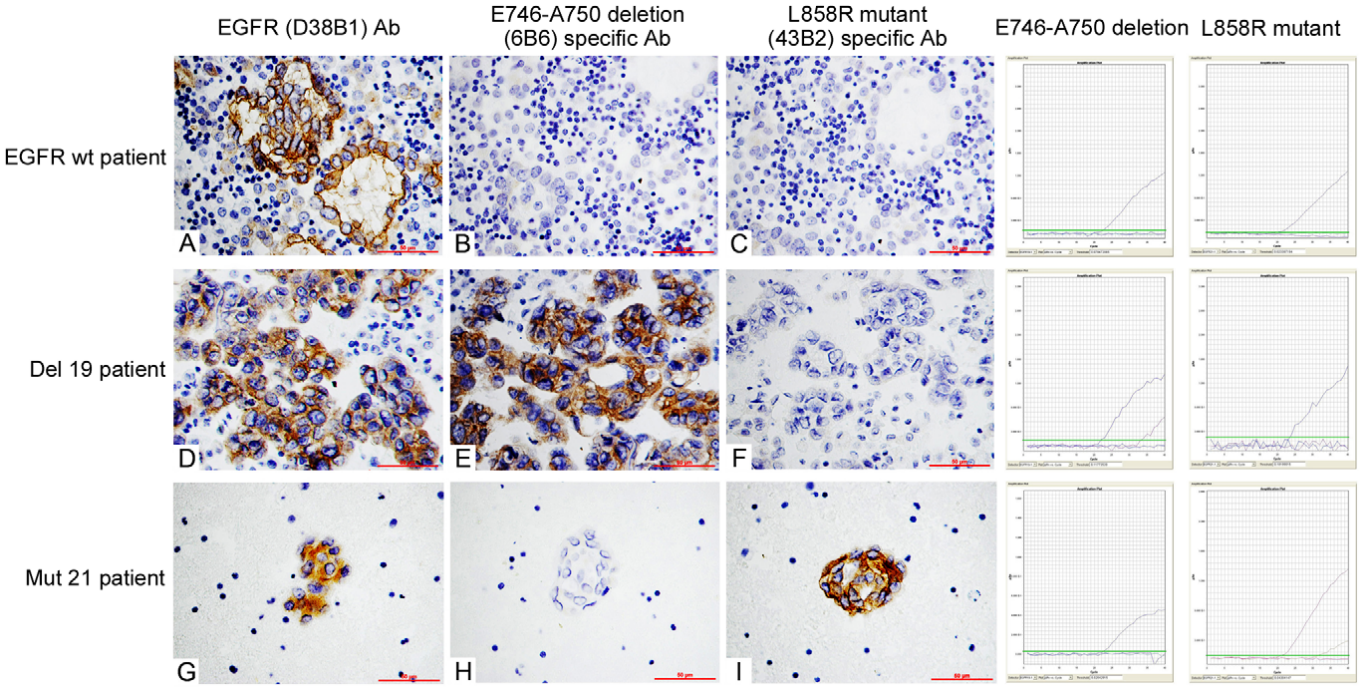
Staining of cytology specimens using EGFR mutation-specific antibodies. The total EGFR protein in cytology samples could be stained with EGFR (D38B1) antibody (A, D and G, 400×). Samples without EGFR mutations were not stained with two mutation-specific antibodies (B and C, 400×). Sample with E746–A750 deletion mutation were stained with E746–A750 deletion (6B6) specific antibody (E, 400×) and samples with L858R mutation were stained with L858R mutant (43B2) specific antibody (I, 400×).

The staining features of cytology specimens using EGFR mutation-specific antibodies were shown in Figure 4. According to our scoring criteria, of the 34 cytology specimens, 15 cases were scored 0, in which 2 cases harbored EGFR mutations and the mutation rate was 13.33% (2/15); 9 cases were scored 1+, in which 4 cases had EGFR mutations and the mutation rate was 44.44% (4/9); 8 cases were scored 2+, in which 4 contained EGFR mutations, and the mutation rate was 50% (4/8); 2 cases were scored 3+, in which 2 cases harbored EGFR mutations, and the mutation rate was 100% (2/2). The detailed results are shown in Table 2.

When score 3+ was considered to be positive, the specificity and PPV in the IHC-based assay was 100% for all the three types of specimens. When score 0 was considered to be negative, the resection specimens resulted in the highest NPV (96.15%), followed by biopsy specimens (92.21%), and cytology specimens had the lowest NPV (88.24%). When score 0 and 1+ were considered to be negative and score 2+ and 3+ considered to be positive, the specificity of resection specimens had the highest NPV (93.02%), followed by biopsy specimens (83.45%), and cytology specimens had the lowest NPV (81.82%)(Table 3).

There were 12 patients with both resection and cytology specimens in our samples, and 6 of them harbored EGFR mutations (3 cases of E746–A750 deletion mutation and 3 cases of L858R point mutation) by molecular-based assay. By IHC-based assay on the resection specimens, 4 of the 6 samples with EGFR mutations were identified as having score of 3+, and the other 2 were identified as having a score of 2+. In contrast, on the cytology specimens, only 1 case had a score of 3+ and 1 case had a score of 2+, and the other 4 cases were all score 1+. In the 6 samples without an EGFR mutation, only 1 case had a score of 1+ by IHC-based assay on the resection specimens, and the other five samples had a score of 0. In contrast, the results on the corresponding cytology specimens included a score of 2+ in 1 case, score 1+ in 1 case, and score 0 in the other 4 cases (Table 4).

**Table 4:** Comparative analyses of IHC-based results between resection and cytology specimens.

Patients	Molecular-based assay	IHC using EGFR mutation-specific antibodies	
	(TaqMan PCR assay)	Resection	Cytology
1	E19 (E746–A750) deletion mutation	3+	3+
2	E19 (E746–A750) deletion mutation	3+	2+
3	E19 (E746–A750) deletion mutation	2+	1+
4	E21 (L858R) point mutation	3+	1+
5	E21 (L858R) point mutation	3+	1+
6	E21 (L858R) point mutation	2+	1+
7	No mutation	1+	0
8	No mutation	0	2+
9	No mutation	0	1+
10	No mutation	0	0
11	No mutation	0	0
12	No mutation	0	0

## Discussion

EGFR mutations can be detected in NSCLC by IHC methods using EGFR mutant-specific antibodies. However, due to the appreciable differences among the criteria for positivity defined by different research groups, the readers or diagnosticians may be confused with or even misled by in regard to a practical application of this method. Therefore, a more extensive research is required to reach a consensus. The results of the present study and other reports [15]–[19], [23] suggest that scoring criteria based on the staining intensity of the membrane and/or cytoplasm of tumor cells and the percentage of the staining area (which was divided into four grades: 0, 1+, 2+ and 3+) may be the best of all available scoring systems. The agreement between IHC-based and molecular-based assays was calculated using Cohen κ in accordance with different levels of immunohistochemical grading. Although the agreement between the 2 testing methods was highest (κ = 0.644) when the score 2+ and 3+ were considered as positive, there were still 33 false-positive cases (32.04%, 33/103) in score 2+ cases and 24 false-negative cases (23.08%, 24/104) in score 1+ cases, resulting in a sensitivity of 77.63%, specificity of 86.64%, PPV of 78.14%, and NPV of 86.4%, none of which was ideal. Hence, with a molecular test as a standard, even if a sample is graded as positive by IHC method, the molecular-based assay may still be needed to verify EGFR mutation status, and thus the screening of EGFR mutations by IHC seems not to be applicable. Even though the number of false-positive cases has been very few in other studies [15]–[19], further molecular detection cannot be entirely abandoned. In this study, we noted that samples with a score of 3+ had a perfect PPV (100%), and score 0 samples had a good NPV (93.06%). If a score of 3+ is considered to be a positive criterion, the patients with positive samples by IHC-based assay could directly receive TKI therapy without need for the verification by a molecular test. The applicability of TKI therapy could be assessed rapidly by immunohistochemistry using EGFR mutation-specific antibodies in nearly 50% of all patients (those scored as 0 or 3+). The chance of mutations in the samples with a score of 1+ was about 23.08%, and a probability of no mutation in them was 76.92%, per our analysis. The rate of mutation in the samples with a score of 2+ was 67.96%, strongly suggesting the possibility of an EGFR mutation in this group. Thereafter, if used properly, screening EGFR mutation status by immunohistochemistry may have a diagnostic value for therapeutic decision making, especially in a medical center that does not have a capacity of molecular testing.

As recommended Wu et al. [22], the expression of total EGFR was also tested in all 399 cases in our study. Unlike the two mutation-specific antibodies, EGFR monoclonal antibody (D38B1) identifies the total EGFR protein regardless of the mutation status. Because the levels of total EGFR in certain cases are very low, the mutant proteins may not be detectable by using two mutation-specific antibodies, even though the cases harbor mutated EGFR gene (as shown in Figure S1). Nonetheless, using the EGFR monoclonal antibody (D38B1) in combination with mutation-specific antibodies likely reduced false-negative results per our analysis. With this approach, the sensitivities of delE746–A750 and L858R mutation-specific antibodies could be increased to 82.56% and 90% respectively. The sensitivity of L858R mutation-specific antibody (43B2) seems higher than delE746–A750 mutation-specific antibody (6B6), which is consistent with what has been reported by Ambrosini-Spaltro A et al [16].

In addition, we have conducted a comparative analysis of the IHC-based results on resection, biopsy and cytology specimens. When a score of 2+ was considered to be positive, the rate of false-positive values for resection specimens was only 17.14% (6/35), whereas the rates of false-positive values for biopsies and cytology specimens were 38.33% (23/60) and 50% (4/8), respectively. This finding suggested that, because of the smaller amount of tissues and tumor cells than resection specimens, the heterogeneity of tumor cells in biopsy or cytology specimens may become more prominent, and thus cause an increased variability of testing results. In addition to the limited number of tumor cells in pleural fluid (cytology specimen), the composition of cellular components was more complex, which often includes many red blood cells, inflammatory cells and mesothelial cells, and thus dilutes the tumor cells. Although cytology specimens can be used for both molecular-based and IHC-based assays, the latter assay tends to show more false-negative values compared with tissue specimens (especially when compared with resection specimens). This may explain a markedly decreased sensitivity of mutant detection in cytology specimens (Table 3). In addition, we have examined 12 cases with both resection and cytology specimens collected. Of these, 6 harbored EGFR mutations by molecular-based assay and the other 6 did not. When score 3+ was used as a threshold, IHC-based assay could identify 4 out of the 6 samples with EGFR mutations in resection specimens, but detect only 1 case in cytology specimens. If score ≥2+ was applied, the assay could identify all the 6 cases with EGFR mutations in resection specimens, but detect only 2 out of the 6 cases in cytology specimens. These results suggest that, with respect to IHC based detection of EGFR mutations, resection specimens were much better than biopsy specimens, and biopsy specimens were significantly better than cytology specimens.

In theory, EGFR proteins on cell membrane or in cytoplasm play different roles in tumor cells and have different interaction with TKIs. However, the treatment effects of TKIs in patients who show a different subcellular distribution of EGFR in tumor cells have not been well investigated yet. As reported previously, we observed that EGFR could locate on tumor cell membrane, in cytoplasm or both on membrane and in cytoplasm by immunohistochemical analysis. In order to determine the functional features of different subcellular distributions in EGFR proteins, we tried to analyze a potential correlation between EGFR mutation status and subcellular distribution of the proteins, but found out no correlation between them. Instead, the staining intensity shows a strong correlation with EGFR mutation status. Therefore, the staining of "the membrane and/or cytoplasm" was equally and jointly considered in our scoring system.

The high activity of EGFR protein in lung cancer is due to the activation mutations, but the gene amplifications may also play a significant role. In this study, 334/399 (83.7%) cases of NSCLC were demonstrated to have elevated expression of EGFR protein (≥2+) by using monoclonal antibody, the rate much higher than standard detection at genetic level (40.6%). It remains to be further investigated if this elevated expression of EGFR in some cases without detectable mutations is mediated by ampification of its gene or other alternative mechanisms. Whether or not the TKIs can be empirically applied to these patients needs to be verified by additional experimental studies and clinical trials.

**Figure 5:**
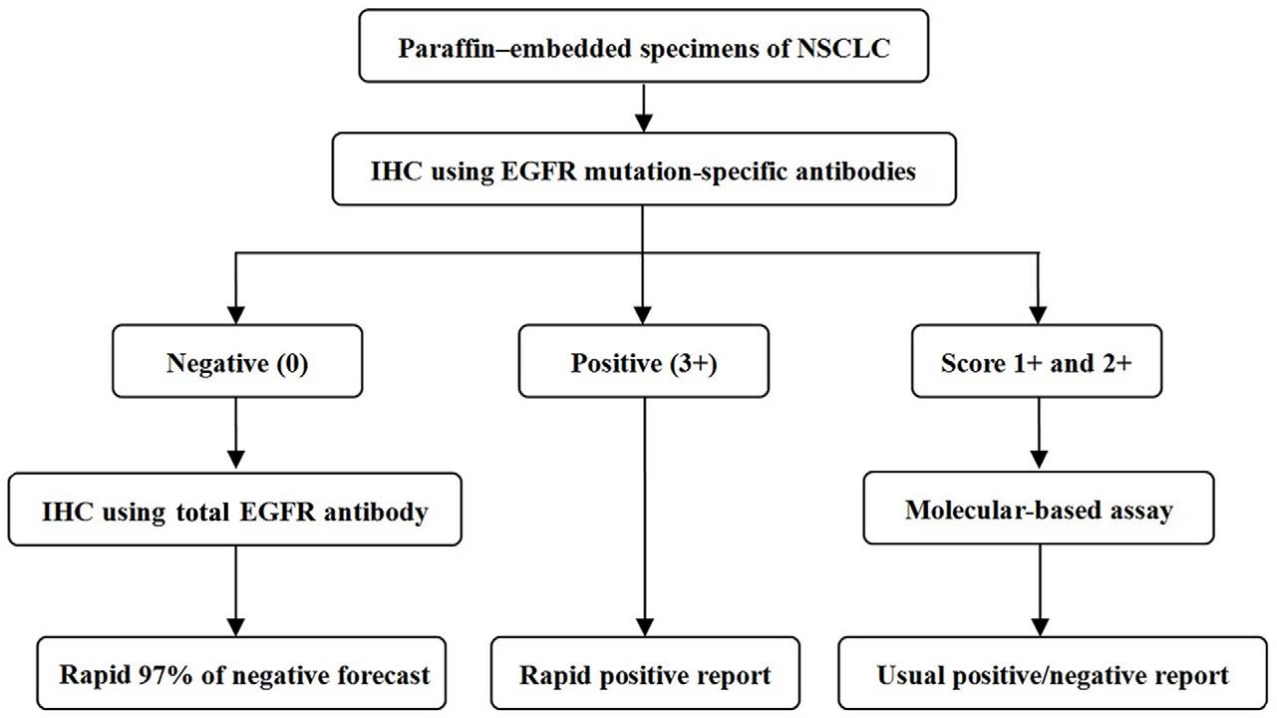
The new flow diagram of screening for EGFR mutations in NSCLC samples. “IHC” refers to immunohistochemical analyses.

In summary, according to our analyses of EGFR mutation status by IHC, patients with a score of 3+ had a perfect PPV (100%), and may accept the TKI treatment directly without need for a confirmatory molecular-based assay. Patients with a score of 0 had a high NPV (93.06%). However, samples with score 1+ or 2+ are unreliable and need further verification of EGFR mutation status by molecular-based assay. Therefore, by using our diagnostic algorithm, in nearly half of all patients (patients with scores 0 or 3+), an applicability of TKI therapy could be determined by immunohistochemistry and more complex, expensive molecular test be avoided. Samples with score 0 in IHC-based assay with the two EGFR mutation-specific antibodies should be further tested for expression of total EGFR using EGFR monoclonal antibody (D38B1), in order to further reduce the false negative rate (Figure 5). Take together, compared with using IHC-based assay alone, this integrated approach combining IHC- and molecular-based assays demonstrates a higher sensitivity (97.37%), specificity (100%) and κ value (0.979),and is thus more practical for the patients screened for a possible TKI therapy. Our diagnostic algorithm may potentially optimize therapeutic options, reduce costs and save time.

## Supporting Information

Figure S1 **The false negative mutation results of IHC due to the low level of total EGFR.** The level of total EGFR in tumor cells was so low (**A** and **D**, 200×) that mutant proteins were undetectable with mutation-specific antibodies in certain cases (**B**, **C**, **E** and **F**, 200×), even though the mutations were identified by molecular-based assay in the cases.
(TIF)

## References

[1] Fukuoka M, Yano S, Giaccone G, Tamura T, Nakagawa K, et al (2003) Multi-institutional randomized phase II trial of gefitinib for previously treated patients with advanced non-small-cell lung cancer (The IDEAL 1 Trial) [corrected]. J Clin Oncol 21: 2237–46. 12748244 10.1200/JCO.2003.10.038

[2] Maemondo M, Inoue A, Kobayashi K, Sugawara S, Oizumi S, et al (2010) Gefitinib or chemotherapy for non-small-cell lung cancer with mutated EGFR. N Engl J Med 362: 2380–8. 20573926 10.1056/NEJMoa0909530

[3] Mok TS, Wu YL, Thongprasert S, Yang CH, Chu DT, et al (2009) Gefitinib or carboplatin-paclitaxel in pulmonary adenocarcinoma. N Engl J Med 361: 947–57. 19692680 10.1056/NEJMoa0810699

[4] Keedy VL, Temin S, Somerfield MR, Beasley MB, Johnson DH, et al (2011) American Society of Clinical Oncology provisional clinical opinion: epidermal growth factor receptor (EGFR) Mutation testing for patients with advanced non-small-cell lung cancer considering first-line EGFR tyrosine kinase inhibitor therapy. J Clin Oncol 29: 2121–7. 21482992 10.1200/JCO.2010.31.8923

[5] Han SW, Kim TY, Hwang PG, Jeong S, Kim J, et al (2005) Predictive and prognostic impact of epidermal growth factor receptor mutation in non-small cell lung cancer patients treated with gefitinib. J Clin Oncol 23: 2493–501. 15710947 10.1200/JCO.2005.01.388

[6] Kim KS, Jeong JY, Kim YC, Na KJ, Kim YH, et al (2005) Predictors of the response to gefitinib in refractory non-small cell lung cancer. Clin Cancer Res 11: 2244–51. 15788673 10.1158/1078-0432.CCR-04-2081

[7] Mitsudomi T, Kosaka T, Endoh H, Horio Y, Hida T, et al (2005) Mutations of the epidermal growth factor receptor gene predict prolonged survival after gefitinib treatment in patients with non-small-cell lung cancer with postoperative recurrence. J Clin Oncol 23: 2513–20. 15738541 10.1200/JCO.2005.00.992

[8] Mitsudomi T, Yatabe Y (2007) Mutations of the epidermal growth factor receptor gene and related genes as determinants of epidermal growth factor receptor tyrosine kinase inhibitors sensitivity in lung cancer. Cancer Sci 98: 1817–24. 17888036 10.1111/j.1349-7006.2007.00607.x PMC11159145

[9] Riely GJ, Politi KA, Miller VA, Pao W (2006) Update on epidermal growth factor receptor mutations in non-small cell lung cancer. Clin Cancer Res 12: 7232–41. 17189394 10.1158/1078-0432.CCR-06-0658

[10] Fukui T, Ohe Y, Tsuta K, Furuta K, Sakamoto H, et al (2008) Prospective study of the accuracy of EGFR mutational analysis by high-resolution melting analysis in small samples obtained from patients with non-small cell lung cancer. Clin Cancer Res 14: 4751–7. 18676744 10.1158/1078-0432.CCR-07-5207

[11] Pao W, Ladanyi M (2007) Epidermal growth factor receptor mutation testing in lung cancer: searching for the ideal method. Clin Cancer Res 13: 49–54–5. 10.1158/1078-0432.CCR-07-1387 17785543

[12] Endo K, Konishi A, Sasaki H, Takada M, Tanaka H, et al (2005) Epidermal growth factor receptor gene mutation in non-small cell lung cancer using highly sensitive and fast TaqMan PCR assay. Lung Cancer 50: 375–384. 16199108 10.1016/j.lungcan.2005.08.009

[13] Zhou C, Ni J, Zhao Y, Su B (2006) Rapid detection of epidermal growth factor receptor mutations in non-small cell lung cancer using real-time polymerase chain reaction with TaqMan-MGB probes. Cancer J 12: 33–39. 16613660 10.1097/00130404-200601000-00007

[14] Kimura H, Fujiwara Y, Sone T, Kunitoh H, Tamura T, et al (2006) High sensitivity detection of epidermal growth factor receptor mutations in the pleural effusion of non-small cell lung cancer patients. Cancer Sci 97: 642–648. 16827805 10.1111/j.1349-7006.2006.00216.x PMC11160100

[15] Yu J, Kane S, Wu J, Benedettini E, Li D, et al (2009) Mutation-specific antibodies for the detection of EGFR mutations in non-small-cell lung cancer. Clin Cancer Res 15: 3023–3028. 19366827 10.1158/1078-0432.CCR-08-2739

[16] Ambrosini-Spaltro A, Campanini N, Bortesi B, Azzoni C, Naldi N, et al (2012) EGFR mutation-specific antibodies in pulmonary adenocarcinoma: a comparison with DNA direct sequencing. Appl Immunohistochem Mol Morphol 20 4: 356–62 doi:10.1097/PAI.0b013e31823e064b. 22710815 10.1097/PAI.0b013e31823e064b

[17] Brevet M, Arcila M, Ladanyi M (2010) Assessment of EGFR mutation status in lung adenocarcinoma by immunohistochemistry using antibodies specific to the two major forms of mutant EGFR. J Mol Diagn 12 2: 169–76. 20093391 10.2353/jmoldx.2010.090140 PMC2871723

[18] Kitamura A, Hosoda W, Sasaki E, Mitsudomi T, Yatabe Y (2010) Immunohistochemical detection of EGFR mutation using mutation-specific antibodies in lung cancer. Clin Cancer Res 16 13: 3349–55. 20570926 10.1158/1078-0432.CCR-10-0129

[19] Kawahara A, Yamamoto C, Nakashima K, Azuma K, Hattori S, et al (2010) Molecular diagnosis of activating EGFR mutations in non-small cell lung cancer using mutation-specific antibodies for immunohistochemical analysis. Clin Cancer Res 16 12: 3163–70. 20423982 10.1158/1078-0432.CCR-09-3239

[20] Kozu Y, Tsuta K, Kohno T, Sekine I, Yoshida A, et al (2011) The usefulness of mutation-specific antibodies in detecting epidermal growth factor receptor mutations and in predicting response to tyrosine kinase inhibitor therapy in lung adenocarcinoma. Lung Cancer 73 1: 45–50. 21129809 10.1016/j.lungcan.2010.11.003

[21] Kato Y, Peled N, Wynes MW, Yoshida K, Pardo M, et al (2010) Novel epidermal growth factor receptor mutation-specific antibodies for non-small cell lung cancer: immunohistochemistry as a possible screening method for epidermal growth factor receptor mutations. J Thorac Oncol 5 10: 1551–8. 20697298 10.1097/JTO.0b013e3181e9da60 PMC2946481

[22] Wu SG, Chang YL, Lin JW, Wu CT, Chen HY, et al (2011) Including total EGFR staining in scoring improves EGFR mutations detection by mutation-specific antibodies and EGFR TKIs response prediction. PLoS One 6 8: e23303. 21858063 10.1371/journal.pone.0023303 PMC3153495

[23] Kawahara A, Taira T, Azuma K, Tominaga M, Hattori S, et al (2012) A diagnostic algorithm using EGFR mutation-specific antibodies for rapid response EGFR-TKI treatment in patients with non-small cell lung cancer. Lung Cancer Oct 78 1: 39–44. 10.1016/j.lungcan.2012.07.002 22858448

[24] Kawahara A, Azuma K, Sumi A, Taira T, Nakashima K, et al (2011) Identification of non-small-cell lung cancer with activating EGFR mutations in malignant effusion and cerebrospinal fluid: rapid and sensitive detection of exon 19 deletion E746-A750 and exon 21 L858R mutation by immunocytochemistry. Lung Cancer 74 1: 35–40. 21444121 10.1016/j.lungcan.2011.02.002

[25] Simonetti S, Molina MA, Queralt C, de Aguirre I, Mayo C, et al (2010) Detection of EGFR mutations with mutation-specific antibodies in stage IV non-small-cell lung cancer. J Transl Med 18 8: 135–42. 10.1186/1479-5876-8-135 PMC3016260 21167064

[26] Dimou A, Agarwal S, Anagnostou V, Viray H, Christensen S, et al (2011) Standardization of epidermal growth factor receptor (EGFR) measurement by quantitative immunofluorescence and impact on antibody-based mutation detection in non-small cell lung cancer. Am J Pathol 179 2: 580–9. 21722621 10.1016/j.ajpath.2011.04.031 PMC3157192

