# Supplementary figures and images for: Ascertaining an Appropriate Diagnostic Algorithm Using EGFR Mutation-Specific Antibodies to Detect EGFR Status in Non-Small-Cell Lung Cancer
Source: PLoS One. 2013 Mar 11;8(3):e59183. doi: 10.1371/journal.pone.0059183 (PMC3594188; doi:10.1371/journal.pone.0059183)

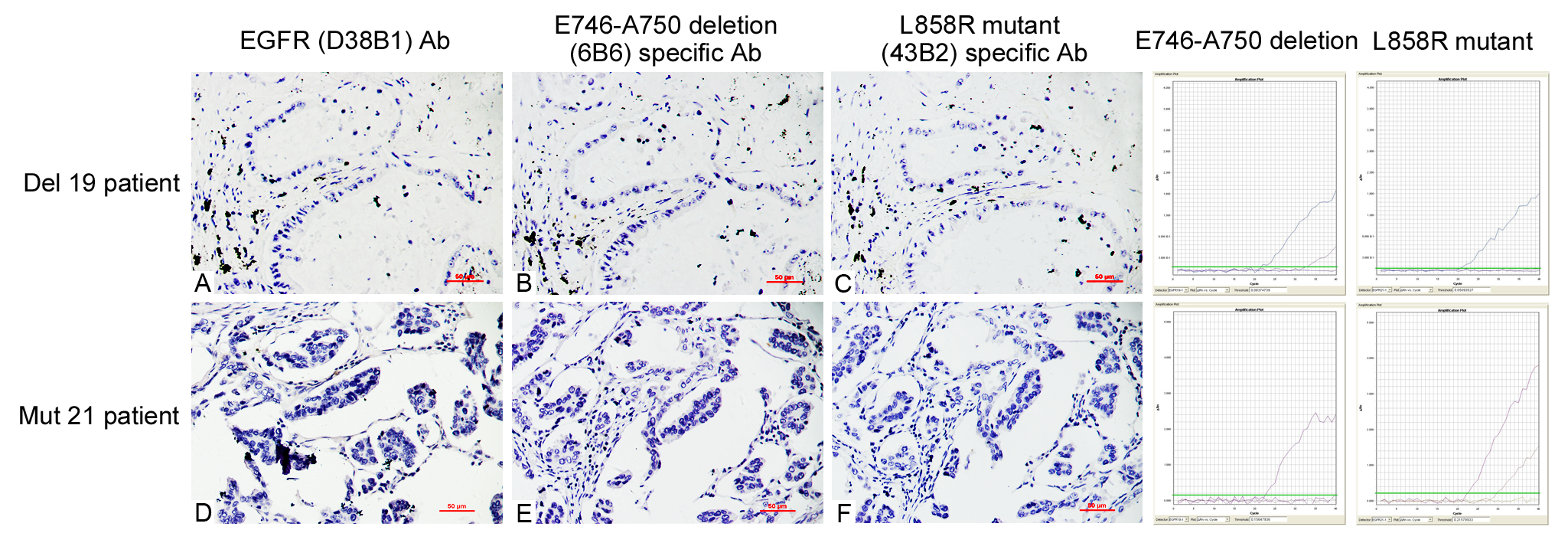

Supplement: Figure S1 — The false negative mutation results of IHC due to the low level of total EGFR. The level of total EGFR in tumor cells was so low (A and D, 200×) that mutant proteins were undetectable with mutation-specific antibodies in certain cases (B, C, E and F, 200×), even though the mutations were identified by molecular-based assay in the cases. (TIF) [file pone.0059183.s001.tif]
